# Functional networks of aging markers in the glomeruli of IgA nephropathy: a new therapeutic opportunity

**DOI:** 10.18632/oncotarget.9033

**Published:** 2016-04-26

**Authors:** Hong Jiang, Ludan Liang, Jing Qin, Yingying Lu, Bingjue Li, Yucheng Wang, Chuan Lin, Qin Zhou, Shi Feng, Shun H. Yip, Feng Xu, EnYin Lai, Junwen Wang, Jianghua Chen

**Affiliations:** ^1^ Kidney Disease Center, The First Affiliated Hospital, College of Medicine, Zhejiang University, P.R. China; ^2^ Kidney Disease Immunology Laboratory, The Third Grade Laboratory, State Administration Of Traditional Chinese Medicine Of China, Hangzhou, P.R. China; ^3^ Key Laboratory Of Multiple Organ Transplantation, Ministry Of Health, Key Laboratory Of Nephropathy, Zhejiang, P.R. China; ^4^ Centre for Genomic Sciences, LKS Faculty of Medicine, The University of Hong Kong, Hong Kong SAR, China; ^5^ School of Life Sciences, The Chinese University of Hong Kong, Hong Kong SAR, China; ^6^ School of Biomedical Sciences, LKS Faculty of Medicine, The University of Hong Kong, Hong Kong SAR, China; ^7^ Department of Physiology, Zhejiang University School of Medicine, Hangzhou, China

**Keywords:** IgAN, glomerular, RNA sequence, aging, networks, Pathology Section

## Abstract

IgA nephropathy(IgAN) is the most common primary glomerular disease in China. Primary infections always occur before IgAN. However, the pathology of IgAN is still unclear. Previously we found that LL37, a protein secreted by senescent cells, was specific for the progression of IgAN, and also played a role in the neutrophil function. So we hypothesized that the infiltration of neutrophils, inflammation factors, and aging markers, which were modulated by functional networks, induced the immune response and renal injury. RNA-Sequencing (RNA-seq) can be used to study the whole transcriptome and detect splicing variants that are expressed in a specific cell type or tissue. We separate glomerulus from the renal biopsy tissues. After RNA extraction, the sequences were analyzed with Illumina HiSeq 2000/2500. 381 genes with differential expression between the IgAN patients and the healthy controls were identified. Only PLAU, JUN, and FOS were related to DNA damage, telomere dysfunction-induced aging markers, neutrophil function and IgA nephropathy. The networks showed the possibility of these genes being connected. We conclude that DNA damage and telomere dysfunction could play important roles in IgA nephropathy. In addition, neutrophils are also important factors in this disease. The networks of these markers showed the mechanism pathways that are involved in the duration of the occurrence and progression of IgA nephropathy and might be a new therapeutic opportunity for disease treatment.

## INTRODUCTION

IgAN is the most common primary glomerular disease in China. The morbidity of IgA nephropathy is 40-50% in primary glomerular disease [1–3]. The characteristic pathological change is the proliferation of mesangial cells and the deposition of the IgA immune complex. There are always primary infections before IgAN [4, 5], but the detailed mechanism is still unclear. In addition, there is no effective treatment for IgA nephropathy. We have discovered a group of specific proteins that were secreted by senescent cells and were induced by telomere dysfunction [6]. This group of proteins is a good predictor of not only human aging but also chronic disease, such as liver fibrosis and myelodysplastic syndrome (MDS). The follow-up studies also showed that among this group of proteins, LL37 is specific for the progression of IgAN [7]. We found higher LL37 expression in renal tissues from IgAN patients, which is also confirmed by IF staining [7]. Interestingly, cells with positive LL37 staining were considered to be neutrophils according to their nuclear shape, which led to the hypothesis that neutrophils may participate in the pathology of IgAN. LL37 not only is related to DNA damage and telomere dysfunction but also plays an important role in the function of the neutrophils [8]. LL37 was very important in the SVV as a neutrophil extracellular traps(NETs) functional factor [9]. LL37 played a novel role in the protection of NETs against degradation by bacterial nucleases [10]. These data suggest some connective links between telomere dysfunction and NETs. These connective links might relate to the pathologic mechanism of IgAN. In this study, we hypothesized that the infiltration of neutrophils, inflammation factors, and aging markers, such as LL37, which were modulated by functional networks, induced the immune response and renal injury. With rapid technological progression, the elucidation of the transcriptome complexity of an organism and understanding the underlying functions of various differentially expressed genes have become a major focus of post-genome research. In addition to the classical microarray approach for profiling transcripts, the recent development of RNA-Sequencing (RNA-seq) has revolutionized the study of whole transcriptomes, providing a potentially unbiased measure of all transcripts and splicing variants that are expressed in a specific cell type [11,12] or tissue [11, 12]. However, the analysis and interpretation of the huge amount of data generated from RNA-Seq pose a practical challenge and demand accurate and easily automated bioinformatics tools for processing datasets [13]. In this study, we detect differentially expressed RNA transcripts between healthy and IgAN patients in aging, inflammation, NETs, and IgAN databases and found the corresponding network, decreasing the cost for the mechanisms that are found, not only in fees, but also in labor. And we could present the bioinformatics proof for the following study of the mechanism's verification.

## RESULTS

### Demographic characteristics of the healthy controls and the IgAN patients

Using RNA-Seq, we analyzed six human glomeruli, three are from the renal biopsy tissues of the transplant donors; these are the healthy controls with pathologic evidence. Other three are from IgAN patients. Table 1 shows the clinical characteristics of the healthy controls and the IgAN patients. The gender, age, urine protein concentration, urine RBC counts, serum ALB, SCr, serum BUN, eGFR (MDRD formula), SBP, DBP, and pathologic diagnosis are listed. IgA nephropathy patients showed higher proteinuria and urine RBC levels and lower eGFR levels. These diagnoses are supported by a pathologic analysis of kidney biopsy tissues.

**Table 1:** Clinical characteristics of 3 healthy controls and 3 IgA nephropathy patients

	Gender	Age	proteinuria	Proteinuria (g/d)	PCr (g/g)	RBC (/HP)	ALB (g/L)	Scr (mmol/L)	BUN (mmol/L)	eGFR (ml/min.kg)	SBP (mmHg)	DBP (mmHg)	Pathologic diagnosis
Control1	F	31	-	0.15	0.25	12.3	38	44	4.8	152.77	112	73	Normal kidney tissue
Control2	F	43	-	0.17	0.19	22.9	38.6	54	4.6	113.06	102	72	Normal kidney tissue
Control3	F	46	-	0.46	0.79	20.7	37	49	4.7	124.8	99	64	Normal kidney tissue
Patient1	M	27	+	0.84	0.12	46.9	39.8	105	5.9	77.53	114	71	IgA Nephropathy
Patient2	M	45	+++	0.88	0.71	+++	48.5	82	5.3	93.25	125	82	IgA Nephropathy
Patient3	F	45	+++	/	/	506.7/++	38.1	47	4.5	131.52	143	95	IgA Nephropathy

Their gender, age, urine protein concentration, urine RBC counts, serum ALB, SCr, serum BUN, eGFR(MDRD formula), SBP, DBP, and pathologic diagnosis are listed above. IgA nephropathy patients showed more proteinuria, urine RBC, and worse eGFR. All those diagnosis were supported by pathologic analysis of kidney biopsy tissues.

### Gene expression analysis by RNA-Seq

To investigate the genes that are changed in IgAN patients, we performed RNA-seq expression screen in the six renal glomeruli. Our bioinformatics analysis of the RNA-seq data revealed a total of 381 genes that were differentially expressed between the IgAN patients and the healthy controls (*q*-value < 0.05). Among them, 229 genes were upregulated, and 152 genes were down regulated in the IgA nephropathy patients compared to the healthy controls (Table S1, Suppl. Figure 1). Thus, our study focused on the hypothesis that modulation by functional networks was induced by the infiltration of aging neutrophils, which caused IgA immune complex deposition and renal injury. We next used the DAVID program to perform enrichment analysis between the differentially expressed genes and gene sets curated from Pubmed search and manual curation. We are particularly interested in genes involved in aging, inflammation, NETs and IgAN.

### Aging transcript expression analysis by RNA-seq

**Figure 1:**
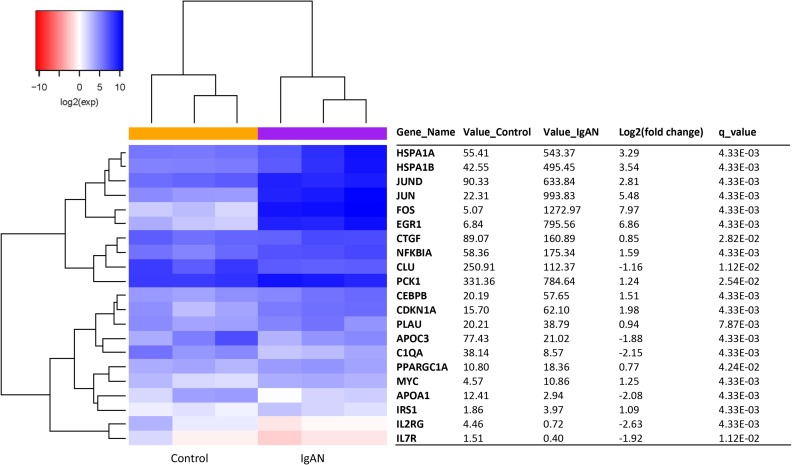
The glomerular expression levels of 21 transcripts correlated with aging The red and blue colors in the heat map depict higher and lower gene expression, respectively. The color intensity indicates the magnitude of the expression differences. A fold change of 2 and a q-value of 0.05 were used as cutoffs. Official names according to the NCBI were given, test values of IgAN and the control are listed, the fold changes indicate the relative alteration of the control value to the IgAN value, and the log2 value of the fold change was used. A positive log2 (fold change) indicates upregulation in the disease group, and a negative log2 (fold change) indicates downregulation.

A total of 21 aging transcripts showed differential expression between the IgAN patients and the healthy controls (*q*-value < 0.05). Of these transcripts, 15 were upregulated, and 6 were downregulated (Figure 1). Red and blue in the heat map depict higher and lower gene expression, respectively. The color intensity indicates the magnitude of the expression differences.

### Inflammation transcript expression analysis by RNA-seq

**Figure 2:**
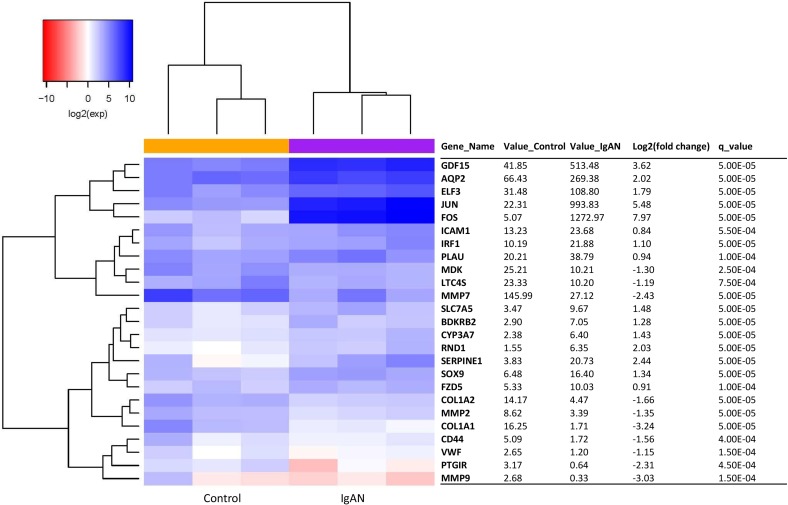
The glomerular expression levels of 25 transcripts correlated with inflammation Refer to Figure 1 for description of the figure.

A total of 25 inflammation transcripts showed differential expression between the IgAN patients and the healthy controls (*q*-value < 0.05). Of these transcripts, 15 were upregulated, and 10 were downregulated (Figure 2). Red and blue in the heat map depict higher and lower gene expression, respectively. The color intensity indicates the magnitude of the expression differences.

### IgA nephropathy transcripts in a glomeruli analysis by RNA-seq

**Figure 3:**
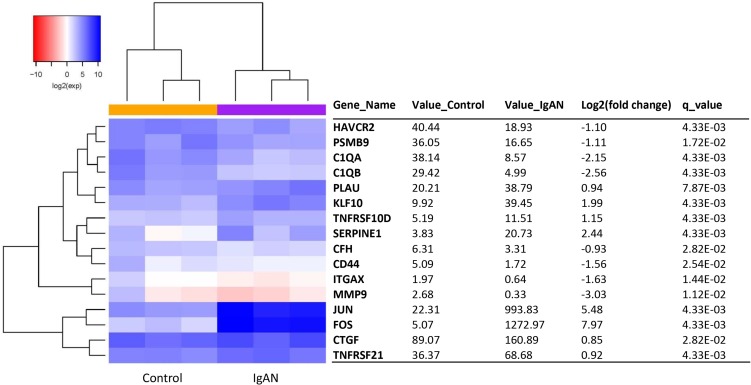
The glomerular expression levels of 16 transcripts that were previously reported as being related to IgA nephropathy Refer to Figure 1 for description of the figure.

A total of 16 IgAN transcripts showed differential expression between the IgAN patients and the healthy controls (*q*-value < 0.05). Of these transcripts, 8 were upregulated, and 8 were downregulated (Figure 3). Red and blue in the heat map depict higher and lower gene expression, respectively. The color intensity indicates the magnitude of the expression differences.

### NETs transcripts in a glomeruli analysis by RNA-seq

**Figure 4:**
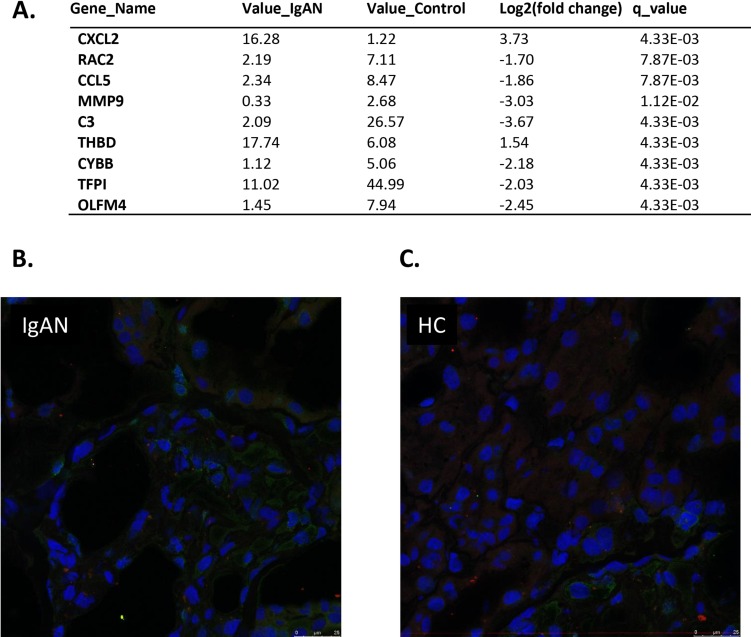
Expression levels of 16 transcripts in glomeruli showed correlation with IgAN **A.** The glomerular expression levels of 9 transcripts correlated with NETs. A fold change of 2 and a q-value of 0.05 were used as cutoffs. Official names according to the NCBI were given, test values of IgAN and the control are listed, the fold change was used to indicate the relative alteration of the control value to the IgAN value, and the log2 value of the fold change was used. A positive log2 (fold change) indicates upregulation in the disease group, and a negative log2 (fold change) indicates downregulation. **B.** and **C.** No neutrophil extracellular traps (NETs) were deposited in kidney biopsies from either the IgA nephropathy patients or the healthy donors. NETs were identified by co-localization with extracellular DNA (blue), the H2A-H2B-complex (green) and LL37 (red). Almost no co-localization of autoantigens was found in the kidney biopsy sections from both populations. IgA: IgA nephropathy patients HD: healthy donars.

A total of 9 NETs transcripts showed differential expression between the IgAN patients and the healthy controls (*q*-value < 0.05). Of these transcripts, 2 were upregulated, while 7 were downregulated (Figure 4A). Red and blue in the heat map depict higher and lower gene expression, respectively. The color intensity indicates the magnitude of the expression differences.

### NETs visualization

We conducted NETs visualization with kidney biopsy tissues from IgA nephropathy patients and healthy donors (Figure 4B, 4C). No neutrophil extracellular traps (NETs) were deposited in the kidney biopsies from either the IgA nephropathy patients or the healthy donors. NETs were identified by co-localization with extracellular DNA (blue), the H2A-H2B-complex (green) and LL37 (red). Almost no co-localization of autoantigen was found in the kidney biopsy sections from both populations.

### Overlap of aging, inflammation, and IgAN related genes

**Figure 5:**
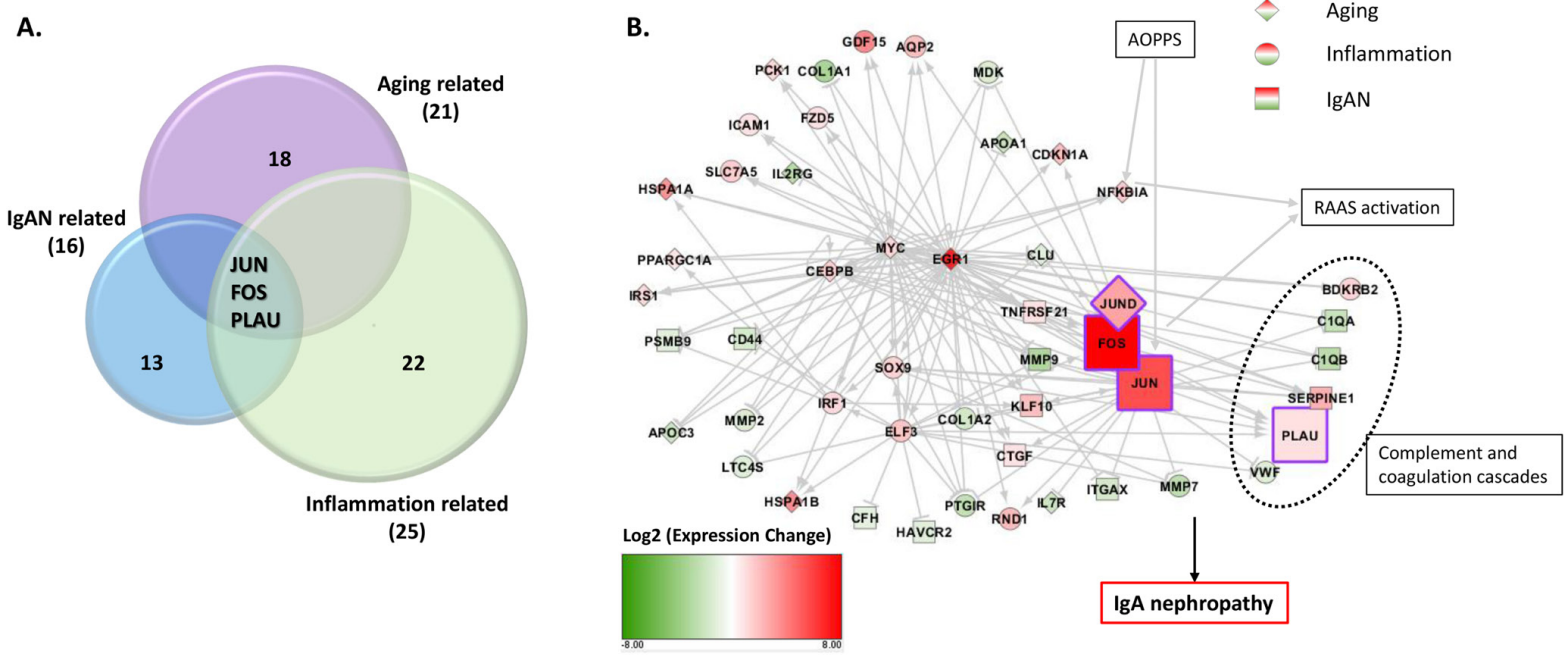
Three transcripts of three genes, JUN, FOS, PLAU, highly expressed in IgAN patients, relatively, were simultaneously participated in aging and inflammation and IgAN **A.** Three transcripts of the genes JUN, FOS, and PLAU were highly expressed in the IgAN patients and were simultaneously included in aging-, inflammation- and IgAN-related genes, respectively. Two of these genes, JUN and FOS could also increase the expression of LL-37. **B.** Network analysis includes differentially expressed genes related with aging(diamond), inflammation(circle) and IgAN(square). Red means up regulation and green means down regulation. Color depth indicates the log2(fold change) value. Lines in grey shows relationship between genes. Arrow means promotion and bar means inhibition. Genes in purple frame were key genes found in our study.

By overlap, we found three genes, JUN, FOS, and PLAU were simultaneously participated in aging and inflammation, and were also previously reported to be related to IgA nephropathy. Two of the three genes, JUN and FOS, could also increase the expression of LL-37 and are therefore indirectly related to NETs (Figure 5A). Figure 5B shows the network of differentially expressed genes related to aging, inflammation and IgAN. They participate in RAAS activation, complement and coagulation cascades.

### Putative mechanisms of aging neutrophil-related IgAN

**Figure 6:**
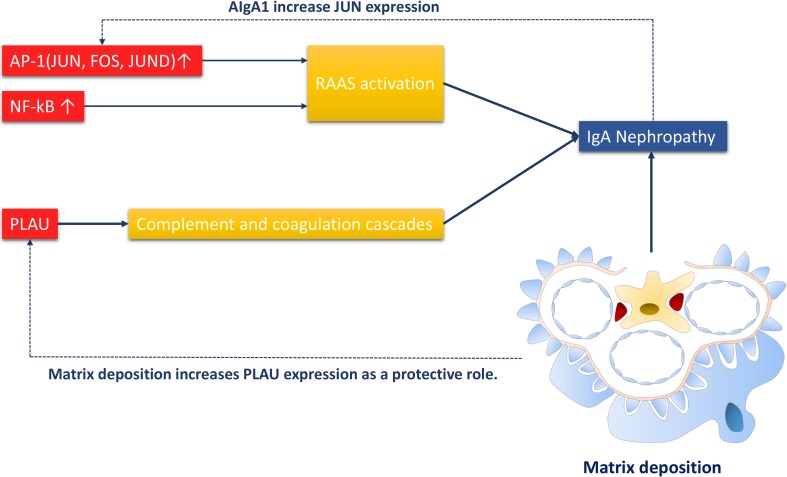
Putative mechanism of of aging neutrophil-related IgAN The RAAS system is activated by JUN, FOS, JUND(components of AP-1 transcription factor) and NF-kappa B, and subsequently induces IgAN. PLAU as a component of complement and coagulation cascades, can also promotes IgAN. Meanwhile, AIgA1 can increase JUN expression. Matrix deposition, an important phenomenon in IgAN can promote PLAU expression as a protective role.

Both JUN and FOS were components of the AP-1 transcription factor. The increased expression of AP-1 and NF-kappa B can both activate the RAAS system, resulting in IgA nephropathy. PLAU is a part in the complement and coagulation cascades, and therefore correlates with IgAN progression. Meanwhile, over-produced AIgA1 could increase JUN expression and thus promoted the disease. Matrix deposition as a characteristic of IgAN could also induce PLAU production in a protective role against disease (Figure 6).

## DISCUSSION

Owning to different cell types and functions, genes differentially expressed between kidney interstitial, tubules and glomerulus. Because IgAN is a glomerular disease, the gene expression variance of the glomerulus should be more obvious and more related to the disease itself. The glomeruli were separated from the renal biopsy tissues, which allowed us to more precisely identify the differentially expressed transcripts specifically to the glomerulus of IgAN. The glomeruli separating techniques were stable and commonly used in mice researches [21, 22]. Because human samples are difficult to obtain, there are no studies on the human glomerulus. In addition, a human glomeruli dissection technique was recently developed. Koitabashi et al. set up a method to isolate human glomeruli from kidney biopsy tissues with further proteomic research [23] in 2011. Bruschi M et al. performed similar studies on LN patients in 2014. Most of these studies focus on method setup and protein inspection; RNA-seq of isolated human glomeruli is rare.

Aging is a biological process characterized by cell senescence and telomere dysfunction [24]. When the cell is stimulated to grow, while the cell cycle is arrested, then the cell becomes senescent (geroconversion) [25]. Irreversible cell cycle arrest happens in response to the telomere dysfunction [26]. Our preliminary study found that LL37 was an aging biomarker induced by DNA damage and telomere dysfunction [6]. LL37 is not only a marker related to IgAN [7] but also an important factor in NETs. Neutrophil infiltration is important in IgAN [27–30]. We hypothesized that the infiltration of the neutrophils, inflammation factors, and aging markers, such as LL37, which were modulated by functional networks, induced the immune response and renal injury. We analyzed the transcriptome and the differentially expressed genes and the genes are significantly enriched in process involved in aging, inflammation, and NETs. RNA-Sequencing techniques [31–33] provide a good way to verify this hypothesis at a low cost. Although previous study found LL37 closely related to IgAN, we did not observe differential RNA expression of LL37 between the healthy controls and the IgAN patients in this transcriptome, maybe because of limited sample size. However, some other aging markers were related to the IgAN patients. Among these 21 aging markers, 3 important aging markers were related to LL37: APOA1, JUN, and FOS. In addition, there was no difference in NETs between the IgAN patients and the healthy controls, suggesting that NETs were not specifically induced in IgAN patients. NETs were specific in some other immune diseases, such as SVV [9] but not IgAN.

PLAU, JUN, and FOS are also related to neutrophil infiltration [34–36]. Both JUN and FOS are components of the AP-1 transcription factor. JUND, though not included in the overlap group, is also a subunit of AP-1. The increased expression of AP-1 activates the RAAS system [34], subsequently resulting in IgA nephropathy. PLAU is a component of complement and coagulation cascades [37]. And complement cascade is closely related with IgAN [38]. Meanwhile, over-produced AIgA1 could increase JUN expression and thus promote disease onset [39]. Matrix deposition, as one of the characteristics of IgAN, can also induce the production of PLAU as a protective role [40].

RNA-Sequence techniques have vastly helped in revising the initial hypothesis, saving money and labor. And we could present the bioinformatics proof for the following study of the mechanism's verification.

There were several limitations in this study. First, because of the difficulty to obtain consent forms, the sample size is limited, only 6 samples were available for analysis with complete and qualified consent forms for a biopsy of kidney tissues. Second, the RNA from the glomerulus is only enough for RNA-Sequencing; with more RNA available, other analyses, such as MicroRNA sequencing, could be simultaneously performed, and the networks could be much more detailed. In this study, the functional networks were induced by the infiltration of aging neutrophils, which caused IgA immune complex deposition and renal injury. This study could help a lot in the follow-up study for detail mechanism's verification. The networks that were correlated with these markers showed the mechanistic pathways that were involved in the occurrence and progression of IgA nephropathy, which might offer a new therapeutic opportunity for disease treatment.

## MATERIALS AND METHODS

### Tissue samples

Studies on human samples were conducted according to the declaration of Helsinki. Three live-donor renal transplant donors supplied the renal biopsy tissues that were used in the transplantations; these tissues were the healthy controls with pathologic evidence. Three IgAN patients supplied the renal biopsy tissues. These renal tissues were collected from Jun. 2011 to Oct. 2011. The dissection procedures were previously published [5]. The glomeruli were dissected at 4°C in an albumin-enriched physiological salt solution (0.1%) using a stereoscopic microscope and sharpened forceps. Tubuli were removed, with the exception of the region of the thick ascending limb of Henle's loop, which touches the glomerulus. The dissection time was limited to 120 min after the biopsies, and the glomeruli were dissected under a microscope (Leica S8AP0).

### RNA quantification and qualification

RNA degradation and contamination were monitored on 1% agarose gels.

RNA purities were checked using the NanoPhotometer^®^ spectrophotometer (IMPLEN, CA, USA).

RNA concentrations were measured using the Qubit^®^ RNA Assay Kit in the Qubit^®^ 2.0 Fluorometer (Life Technologies, CA, USA).

RNA integrities were assessed using the RNA Nano 6000 Assay Kit of the Agilent Bioanalyzer 2100 system (Agilent Technologies, CA, USA).

### Library preparation for sequencing

A total of 3 μg of RNA per sample was used as the input material for the RNA sample preparations. Sequencing libraries were generated using the NEBNext^®^ Ultra™ RNA Library Prep Kit for Illumina^®^ (NEB, USA) following the manufacturer's recommendations, and index codes were added to attribute sequences to each sample. Briefly, mRNA was purified from the total RNA using poly-T oligo-attached magnetic beads. Fragmentation was carried out using divalent cations under an elevated temperature in NEBNext First Strand Synthesis Reaction Buffer (5×). First-strand cDNA was synthesized using a random hexamer primer and M-MuLV Reverse Transcriptase (RNaseH-). Second-strand cDNA synthesis was subsequently performed using DNA Polymerase I and RNase H. The remaining overhangs were converted into blunt ends via exonuclease/polymerase activities. After the adenylation of the 3′ ends of the DNA fragments, the NEBNext Adaptor with a hairpin loop structure was ligated to prepare for hybridization. To select cDNA fragments of preferentially 150~200 bp in length, the library fragments were purified with the AMPure XP system (Beckman Coulter, Beverly, USA). Then, 3 μl of USER Enzyme (NEB, USA) was used with size-selected, adaptor-ligated cDNA at 37°C for 15 min followed by 5 min at 95°C before PCR. Then, PCR was performed with Phusion High-Fidelity DNA polymerase, Universal PCR primers and Index (X) Primer. Finally, the PCR products were purified (AMPure XP system), and the library quality was assessed on the Agilent Bioanalyzer 2100 system.

### Clustering and sequencing (novogene experimental department)

The clustering of the index-coded samples was performed on a cBot Cluster Generation System using the TruSeq SR Cluster Kit v3-cBot-HS (Illumina) according to the manufacturer's instructions. After the cluster generation, the library preparations were sequenced on an Illumina HiSeq 2000/2500 platform, and 150 bp/100 bp/50 bp paired/single-end reads were generated.

### Statistics

The quality of raw sequencing reads was checked with FastQC [14]. Sequencing adapter sequences at the two ends of raw reads were cleaned by Trimmomatic [15]. Then, the cleaned reads were mapped to the human genome hg19 by STAR [16]. The gene expression was quantified, and the differentially expressed genes (DEGs) were identified by Cuffdiff 2 [17] with the *q*-value < 0.05. Functional annotation and pathway enrichment analysis were performed with DAVID [18]. Polysearch2 [19], web-server that supports text mining by integrating millions of scientific literatures from Medline and Pubmed as well as other databases to ensure a more accurate prediction, was used to search for inflammation/aging-related gene relationships. In the study, we used the gene as the query to find inflammation/aging related genes. In the advanced search options, the synonym list was default from the tool. For example, the filter words was customized using inflammation related keywords (inflammation, inflammatory, inflamed, pro-inflammatory, anti-inflammatory) to extract inflammation-related genes. A relevance score was calculated from the associations after the extraction, and later converted to Z score. Associated genes with z score above 1 were selected for subsequent analysis. IgA nephropathy related genes and proteins were searched through the PubMed database(Table S2).

### Visualization of NETs

Double immunofluorescence staining was conducted on frozen sections of kidney biopsies from IgA nephropathy patients(*n* = 3) and healthy donors(*n* = 3). Slides were blocked and permeabilized with phosphate buffered saline containing 2% BSA (Sigma, Germany) and 0.2% Triton X-100 (Sigma, Germany) for 15 min. The incubation of primary antibodies, mouse monoclonal anti-DNA/Histone 1 (1:500; Millipore, America) and rabbit anti-LL37 [20] was carried out overnight at 4 Celsius degree. Then, the samples were incubated with Alexa-Fluor-488-labeled goat-anti-mouse antibody (1:500; Invitrogen, UK) and Alexa-Fluor-633-labeled goat-anti-rabbit antibody (1:500; Invitrogen, UK). The mounted samples were viewed with an ICS SP8 confocal laser scanning microscope, and the intensity of NETs and LL37 was calculated using LAS-AF software.

## SUPPLEMENTARY MATERIAL FIGURES AND TABLES


