# Supplementary material for: Functional networks of aging markers in the glomeruli of IgA nephropathy: a new therapeutic opportunity
Source: Oncotarget. 2016 Apr 26;7(23):33616–26. doi: 10.18632/oncotarget.9033 (PMC5085107; doi:10.18632/oncotarget.9033)
Supplement: Supplementary file 1 [file oncotarget-07-33616-s001.pdf]

Functional networks of aging markers in the glomeruli of IgA nephropathy: a new therapeutic opportunity

Supplementary Material

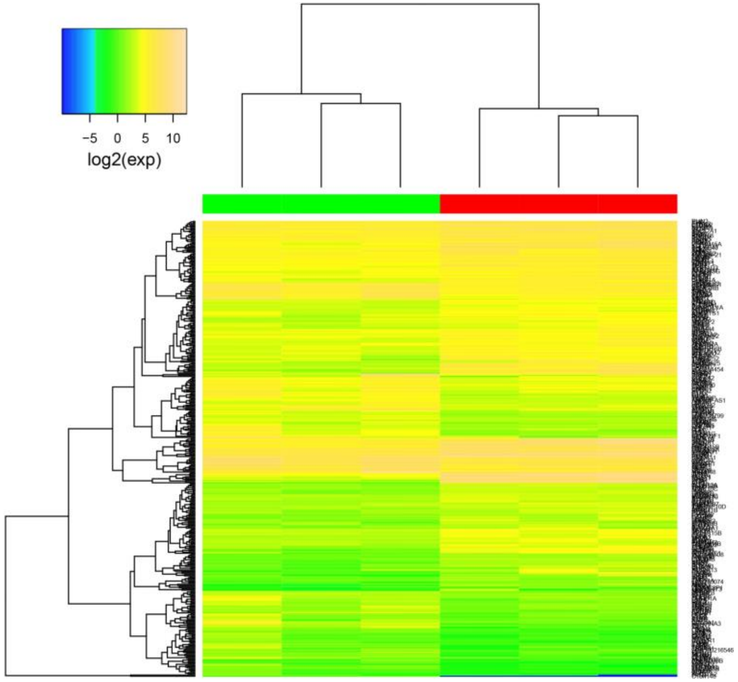

Figure S1. Heatmap of 381 differentially expressed genes between IgAN and Healthy controls.

381 genes differentially expressed between IgAN and Healthy control. Among them, 229 genes were up regulated and 152 genes were down regulated in IgAN group respectively. The color intensity indicates the magnitude of the expression differences according to Log2 value. A fold change of 2 and a q-value of 0.05 were used as cutoffs. Official names according to the NCBI were given.

Table S1. Genes differentially expressed between IgAN and Healthy Control.

381 genes differentially expressed between two groups. 229 genes were up regulated and 152 were down regulated in IgAN groups respectively. Gene Name, Gene ID, and gene locus in chromosome were given according to NCBI. Value of IgAN and healthy control of each gene were shown. Log2 value of IgAN/Control were used to show the difference. p_value and q_value were used to show the significance.

Gene Name	Gene ID	Locus	value_IgAN	value-Control	Log2(IgAN/Control)	p_value	q_value
FOS	NM_005252	chr14:75745480-75748937	1272.97	5.07	7.971649842	5.00E-05	4.33E-03
ATF3	NM_001674	chr1:212738675-212794119	284.592	1.46859	7.598319616	5.00E-05	0.00433264
EGR1	NM_001964	chr5:137801180-137805004	795.563	6.84126	6.86157031	5.00E-05	4.33E-03
NR4A1	NM_173157	chr12:52416615-52453291	524.25	9.1814	5.83539699	5.00E-05	0.00433264
DUSP1	NM_004417	chr5:172195092-172198203	702.076	15.7114	5.481743565	5.00E-05	0.00433264
JUN	NM_002228	chr1:59246462-59249785	993.83	22.31	5.47693779	5.00E-05	4.33E-03
ZFP36	NM_003407	chr19:39897486-39900052	751.294	27.2675	4.784123242	5.00E-05	0.00433264
KLF4	NM_004235	chr9:110247132-110252047	105.373	3.94703	4.738593954	5.00E-05	0.00433264
JUNB	NM_002229	chr19:12902309-12904125	833.657	31.3491	4.732957993	5.00E-05	0.00433264
NR4A3	NM_006981	chr9:102584136-102629173	5.13018	0.200631	4.676393003	5.00E-05	0.00433264
HSPA6	NM_002155	chr1:161494035-161496687	24.1604	1.17744	4.35891889	5.00E-05	0.00433264
BTG2	NM_006763	chr1:203274663-203278729	334.248	17.7377	4.236028071	5.00E-05	0.00433264
NR4A2	NM_006186	chr2:157180943-157189287	5.59635	0.299352	4.224571378	5.00E-05	0.00433264
CLDN14	NM_012130	chr21:37832919-37948867	26.4052	1.55907	4.082064459	5.00E-05	0.00433264
SOCS3	NM_003955	chr17:76352857-76356160	143.023	8.48394	4.075368942	5.00E-05	0.00433264
PDK4	NM_002612	chr7:95212808-95225925	90.5654	5.69776	3.990493223	5.00E-05	0.00433264
LOC284454	NR_036515	chr19:13945329-13947173	87.5004	6.34902	3.784683784	5.00E-05	0.00433264
CXCL2	NM_002089	chr4:74962753-74964997	16.2835	1.22317	3.734713995	5.00E-05	0.00433264

ERRFI1	NM_018948	chr1:8071778-8086393	130.531	9.86058	3.726576157	5.00E-05	0.00433264
RASD1	NM_016084	chr17:17397752-17399709	115.032	9.01738	3.673183121	5.00E-05	0.00433264
SIK1	NM_173354	chr21:44834397-44847002	37.4594	2.96486	3.659291904	5.00E-05	0.00433264
GDF15	NM_004864	chr19:18496967-18499986	513.477	41.8541	3.616858796	5.00E-05	4.33E-03
ZBTB16	NM_006006	chr11:113930430-114121397	21.867	1.7965	3.605494466	1.00E-04	0.00787453
ITLN1	NM_017625	chr1:160846329-160854960	12.1498	1.02423	3.568320939	5.00E-05	0.00433264
HSPA1B	NM_005346	chr6_qbl_hap6:3089162-3091686	495.447	42.549	3.541533604	5.00E-05	4.33E-03
DUSP2	NM_004418	chr2:96808907-96811179	8.1702	0.706089	3.532449448	5.00E-05	0.00433264
CSRNP1	NM_033027	chr3:39183341-39195102	74.9919	7.03298	3.414526756	5.00E-05	0.00433264
SLC25A25	NM_052901	chr9:130830478-130871537	82.8393	7.81718	3.4055952	5.00E-05	0.00433264
HSPA1A	NM_005345	chr6_qbl_hap6:3076937-3079366	543.369	55.405	3.293844177	5.00E-05	4.33E-03
CYR61	NM_001554	chr1:86046443-86049648	204.445	21.1991	3.269637858	5.00E-05	0.00433264
IER2	NM_004907	chr19:13261281-13265718	579.833	60.5322	3.259862753	5.00E-05	0.00433264
LINC00473	NR_026860	chr6:166337535-166403103	3.80195	0.420773	3.175625521	4.50E-04	0.0281556
KLF6	NM_001300	chr10:3818187-3827473	224.364	25.029	3.164168653	5.00E-05	0.00433264
FKBP5	NM_004117	chr6:35541361-35704724	55.9783	6.3077	3.149681724	5.00E-05	0.00433264
PPP1R15A	NM_014330	chr19:49375648-49379319	175.171	20.929	3.065188667	5.00E-05	0.00433264
GADD45B	NM_015675	chr19:2476122-2478257	290.542	35.7512	3.022683159	5.00E-05	0.00433264
ARC	NM_015193	chr8:143692404-143695833	6.13515	0.763615	3.00618127	5.00E-05	0.00433264
DNAJB1	NM_006145	chr19:14625581-14629201	555.867	73.851	2.912050375	5.00E-05	0.00433264
RHOB	NM_004040	chr2:20646834-20649201	887.099	120.247	2.883094217	5.00E-05	0.00433264
SLC2A3	NM_006931	chr12:8071823-8088892	10.9006	1.51111	2.850726958	5.00E-05	0.00433264
JUND	NM_005354	chr19:18390503-18392466	633.84	90.33	2.810794158	5.00E-05	4.33E-03
KLF2	NM_016270	chr19:16435650-16438339	427.777	62.0176	2.786109314	5.00E-05	0.00433264
PER1	NM_002616	chr17:8043787-8055753	77.536	11.3539	2.77167837	5.00E-05	0.00433264

NFKBIZ	NM_031419	chr3:101498028-101579869	22.1377	3.31126	2.74105314	5.00E-05	0.00433264
RGS1	NM_002922	chr1:192544856-192549159	6.8355	1.09587	2.640970206	5.00E-05	0.00433264
TSC22D3	NM_004089	chrX:106956451-107019017	197.296	33.1006	2.575432434	5.00E-05	0.00433264
BHLHE40	NM_003670	chr3:5021096-5026865	170.876	30.1023	2.505004154	5.00E-05	0.00433264
SERPINE1	NM_000602	chr7:100770369-100782547	20.73	3.83	2.437807314	5.00E-05	4.33E-03
FAM46B	NM_052943	chr1:27331510-27339333	13.8443	2.59076	2.41784483	5.00E-05	0.00433264
TRIB1	NM_025195	chr8:126442562-126450647	38.0166	7.12222	2.416230606	5.00E-05	0.00433264
LDLR	NM_000527	chr19:11200037-11244505	14.6007	2.85338	2.355293741	5.00E-05	0.00433264
IGFBP1	NM_000596	chr7:45927958-45933267	5.94834	1.17597	2.338635856	1.00E-04	0.00787453
F2RL3	NM_003950	chr19:16999825-17002830	6.62782	1.36048	2.284418674	5.00E-05	0.00433264
USP2	NM_171997	chr11:119225924-119252436	102.212	21.9352	2.220244817	5.00E-05	0.00433264
CEBPD	NM_005195	chr8:48649475-48650726	443.694	95.6473	2.213768895	5.00E-05	0.00433264
C5orf27	NR_026936	chr5:95187935-95195836	11.1726	2.52817	2.143799576	5.00E-05	0.00433264
SNAI1	NM_005985	chr20:48599512-48605420	6.2025	1.42713	2.119733071	5.00E-05	0.00433264
TOB1	NM_005749	chr17:48939586-48945732	99.0969	23.1865	2.095554865	5.00E-05	0.00433264
DNAJB4	NM_007034	chr1:78470635-78482995	37.0804	8.69054	2.093139078	5.00E-05	0.00433264
ZDHHC8P1	NR_003950	chr22:23732791-23744799	1.87839	0.440316	2.092885459	8.00E-04	0.0441813
IL1RL1	NM_003856	chr2:102927961-102968497	65.1635	15.4904	2.072689696	1.00E-04	0.00787453
RND1	NM_014470	chr12:49250915-49259653	6.34603	1.55144	2.032246438	5.00E-05	4.33E-03
AQP2	NM_000486	chr12:50344523-50359465	269.384	66.4314	2.019726941	5.00E-05	4.33E-03
G6PC	NM_000151	chr17:41052813-41066450	118.992	29.591	2.007634225	5.00E-05	0.00433264
KLF10	NM_005655	chr8:103661004-103668130	39.45	9.92	1.991318997	5.00E-05	4.33E-03
CDKN1A	NM_000389	chr6:36644236-36655116	62.1037	15.7045	1.983501212	5.00E-05	4.33E-03
C2CD4B	NM_001007595	chr15:62455736-62457482	6.37671	1.63521	1.963336348	5.00E-05	0.00433264
FOSL2	NM_005253	chr2:28607275-28637516	53.6349	13.9563	1.942255547	5.00E-05	0.00433264

RGS3	NM_144489	chr9:116207008-116360023	61.6578	16.1764	1.930392837	5.00E-05	0.00433264
DUSP6	NM_001946	chr12:89741601-89746636	36.9203	10.0721	1.874049764	5.00E-05	0.00433264
DUSP5	NM_004419	chr10:112257624-112271302	15.7042	4.32783	1.859434716	5.00E-05	0.00433264
LEAP2	NM_052971	chr5:132209357-132210582	14.3402	4.01006	1.838369417	2.50E-04	0.017201
HBEGF	NM_001945	chr5:139712427-139726188	18.2622	5.1631	1.822551128	5.00E-05	0.00433264
FAM83D	NM_030919	chr20:37554954-37581703	5.82149	1.66066	1.809631726	5.00E-05	0.00433264
ELF3	NM_004433	chr1:201979689-201986315	108.798	31.4801	1.789140007	5.00E-05	4.33E-03
CLDN4	NM_001305	chr7:73245192-73247023	202.156	58.7805	1.782059487	5.00E-05	0.00433264
KLF11	NM_003597	chr2:10183681-10194963	11.5234	3.37131	1.773185252	5.00E-05	0.00433264
OVOL1	NM_004561	chr11:65554504-65564690	16.8495	4.93905	1.770400302	5.00E-05	0.00433264
RGS16	NM_002928	chr1:182567757-182573548	8.83178	2.60311	1.762467958	5.00E-05	0.00433264
CHAC1	NM_024111	chr15:41245635-41248717	5.95207	1.75622	1.760917912	1.50E-04	0.0111745
NUAK2	NM_030952	chr1:205271190-205290883	37.0247	11.3945	1.700150426	5.00E-05	0.00433264
FAM150B	NM_001002919	chr2:279560-288308	11.0153	3.39596	1.697617416	5.00E-05	0.00433264
PLK3	NM_004073	chr1:45266035-45272957	9.59836	2.97987	1.687538531	5.00E-05	0.00433264
CCNL1	NM_020307	chr3:156865585-156878482	48.1718	15.0642	1.677064774	5.00E-05	0.00433264
RIPK4	NM_020639	chr21:43159528-43187249	40.2973	12.6118	1.675908982	5.00E-05	0.00433264
ITPRIP	NM_001272013	chr10:106069453-106098251	9.19313	2.88846	1.670255626	5.00E-05	0.00433264
ZC3H12A	NM_025079	chr1:37940118-37949978	10.8801	3.42419	1.667857156	5.00E-05	0.00433264
ZNF165	NM_003447	chr6:28048481-28057340	4.99815	1.57743	1.663818213	1.00E-04	0.00787453
CHGB	NM_001819	chr20:5891973-5906005	5.42826	1.78743	1.60260308	5.00E-05	0.00433264
ZYG11A	NM_001004339	chr1:53308182-53360247	4.09817	1.3548	1.596899939	5.00E-05	0.00433264
KLF9	NM_001206	chr9:72999512-73029573	35.0312	11.5961	1.595000729	5.00E-05	0.00433264
MIDN	NM_177401	chr19:1248551-1259142	74.5108	24.688	1.593639586	5.00E-05	0.00433264
NFKBIA	NM_020529	chr14:35870715-35873960	175.34	58.3599	1.587105837	5.00E-05	4.33E-03

HLA-H	NR_001434	chr6_ssto_hap7:1186893-1190196	51.906	17.3198	1.583479044	5.00E-05	0.00433264
PIM1	NM_002648	chr6:37137921-37143204	36.3048	12.2102	1.572073474	5.00E-05	0.00433264
LOC285074	NR_026846	chr2:87257797-87303536	3.1698	1.07789	1.556181859	5.50E-04	0.0327313
RNF152	NM_173557	chr18:59482303-59560304	36.828	12.5537	1.552690414	5.00E-05	0.00433264
IER3	NM_003897	chr6_ssto_hap7:2043292-2044644	127.693	43.603	1.550180135	5.00E-05	0.00433264
THBD	NM_000361	chr20:23026269-23030301	17.7383	6.08026	1.54466283	5.00E-05	0.00433264
NR0B2	NM_021969	chr1:27237974-27240567	18.1532	6.26428	1.535003281	5.00E-05	0.00433264
CEBPB	NM_001285878	chr20:48807119-48809227	57.6484	20.1911	1.513561057	5.00E-05	4.33E-03
SLCO4A1	NM_016354	chr20:61273796-61303647	8.7969	3.08157	1.513329649	5.00E-05	0.00433264
TNS4	NM_032865	chr17:38632079-38657854	2.69265	0.946769	1.507942342	2.00E-04	0.0143819
SDPR	NM_004657	chr2:192699031-192712006	25.182	8.95759	1.49121033	5.00E-05	0.00433264
PPP1R15B	NM_032833	chr1:204372491-204380944	22.1704	7.89117	1.490323674	5.00E-05	0.00433264
SLC7A5	NM_003486	chr16:87863628-87903100	9.66792	3.47084	1.477920674	5.00E-05	4.33E-03
SGK1	NM_005627	chr6:134490383-134639196	138.266	50.0559	1.465834405	5.00E-05	0.00433264
GADD45G	NM_006705	chr9:92219926-92221469	49.769	18.0698	1.461666861	5.00E-05	0.00433264
UPP2	NM_173355	chr2:158851690-158992666	26.5563	9.72669	1.449033311	5.00E-05	0.00433264
SLC7A13	NM_138817	chr8:87226287-87242604	12.9765	4.76082	1.446619326	5.00E-05	0.00433264
LOC340508	NR_002942	chr9:99837952-99844227	5.00295	1.84191	1.441576464	1.00E-04	0.00787453
GADD45A	NM_001924	chr1:68150859-68154021	259.099	95.3934	1.441542089	5.00E-05	0.00433264
STC1	NM_003155	chr8:23699433-23712320	12.4871	4.6039	1.439510062	5.00E-05	0.00433264
SLC12A1	NM_000338	chr15:48498497-48596275	144.531	53.4939	1.433932672	5.00E-05	0.00433264
CYP3A7	NM_000765	chr7:99282301-99332819	6.39667	2.37995	1.426389793	5.00E-05	4.33E-03
KCNK5	NM_003740	chr6:39156746-39197251	107.75	40.1273	1.425031878	5.00E-05	0.00433264
TSKU	NM_015516	chr11:76493356-76509198	110.835	41.3969	1.420818893	5.00E-05	0.00433264
TMEM252	NM_153237	chr9:71151497-71155783	27.4002	10.4975	1.384140636	5.00E-05	0.00433264

CXCR7	NM_020311	chr2:237478379-237490994	7.62303	2.92417	1.382337367	1.50E-04	0.0111745
HSPA2	NM_021979	chr14:65007185-65009954	40.7047	15.9045	1.355760368	5.00E-05	0.00433264
TSPYL2	NM_022117	chrX:53111541-53117728	36.0162	14.1341	1.349465952	5.00E-05	0.00433264
SOX9	NM_000346	chr17:70117160-70122560	16.3961	6.4753	1.340333755	5.00E-05	4.33E-03
RNF186	NM_019062	chr1:20140521-20141771	126.618	50.0393	1.339349	5.00E-05	0.00433264
SMAD7	NM_005904	chr18:46446222-46477081	15.3172	6.08746	1.331240303	8.00E-04	0.0441813
TGIF1	NM_003244	chr18:3411924-3458406	37.7828	15.1179	1.321471869	5.00E-05	0.00433264
BAG3	NM_004281	chr10:121410881-121437329	93.9734	37.9012	1.31000892	5.00E-05	0.00433264
NFIL3	NM_005384	chr9:94171326-94186908	21.7112	8.85275	1.294241882	1.00E-04	0.00787453
TICAM1	NM_182919	chr19:4815935-4831754	21.1275	8.63719	1.290488133	5.00E-05	0.00433264
BDKRB2	NM_000623	chr14:96671134-96710666	7.04906	2.90351	1.279632883	5.00E-05	4.33E-03
CISH	NM_145071	chr3:50643884-50649262	60.9095	25.1719	1.274853145	5.00E-05	0.00433264
CITED2	NM_006079	chr6:139693391-139695787	179.491	74.7465	1.263833575	5.00E-05	0.00433264
TUFT1	NM_001126337	chr1:151512780-151556059	19.7419	8.29188	1.251489701	5.00E-05	0.00433264
MYC	NM_002467	chr8:128748314-128753680	10.8615	4.57028	1.248868896	5.00E-05	4.33E-03
PCK1	NM_002591	chr20:56136136-56141513	784.64	331.356	1.243648841	4.00E-04	2.54E-02
PNRC1	NM_006813	chr6:89790428-89794879	111.503	47.2771	1.237869079	5.00E-05	0.00433264
SERTAD1	NM_013376	chr19:40928408-40931932	51.0519	21.7323	1.232123787	5.00E-05	0.00433264
CCRN4L	NM_012118	chr4:139936912-139967093	10.653	4.55791	1.224815423	5.00E-05	0.00433264
RRAD	NM_004165	chr16:66955581-66959439	23.4599	10.0479	1.223302853	1.00E-04	0.00787453
PROB1	NM_001161546	chr5:138727634-138730885	5.2128	2.23824	1.219693768	2.50E-04	0.017201
TSC22D2	NM_014779	chr3:150126787-150177615	15.1445	6.56231	1.206518295	5.00E-05	0.00433264
RASL11B	NM_023940	chr4:53728494-53733002	10.8646	4.71747	1.203549812	2.50E-04	0.017201
SOWAHB	NM_001029870	chr4:77816081-77819002	7.87371	3.42043	1.202865872	5.00E-05	0.00433264
CBX4	NM_003655	chr17:77806954-77813213	15.6609	6.80815	1.201832395	5.00E-05	0.00433264

MST1P2	NR_027504	chr1:16972068-16976915	22.2123	9.69258	1.196406143	5.00E-05	0.00433264
C1orf51	NM_144697	chr1:150255228-150259501	7.84608	3.43001	1.193759263	5.00E-04	0.0306361
TPPP	NM_007030	chr5:659976-693510	9.35069	4.10106	1.189075976	5.00E-05	0.00433264
PLK2	NM_006622	chr5:57749809-57755966	13.9245	6.12962	1.183755981	5.00E-05	0.00433264
ZFAND2A	NM_182491	chr7:1192542-1199855	34.478	15.2947	1.17264428	5.00E-05	0.00433264
FAM110C	NM_001077710	chr2:38813-46588	8.33195	3.70779	1.168094646	5.00E-05	0.00433264
HMGCS2	NM_005518	chr1:120290618-120311555	27.6308	12.3739	1.158977053	5.00E-04	0.0306361
TNFRSF10D	NM_003840	chr8:22993100-23021543	11.51	5.19	1.149155031	5.00E-05	4.33E-03
FMO2	NM_001460	chr1:171154387-171181822	3.26902	1.48282	1.140514724	3.00E-04	0.0198498
ERF	NM_006494	chr19:42751716-42759309	130.17	59.8281	1.12150184	5.00E-05	0.00433264
DDIT4	NM_019058	chr10:74033676-74035797	64.7425	29.791	1.119836524	5.00E-05	0.00433264
TMEM207	NM_207316	chr3:190146443-190167665	12.3485	5.69246	1.117211651	6.50E-04	0.0378132
RORC	NM_005060	chr1:151778546-151804348	32.6624	15.0746	1.115511081	2.50E-04	0.017201
SRF	NM_003131	chr6:43138919-43149244	28.7005	13.3444	1.104841431	5.00E-05	0.00433264
IRF1	NM_002198	chr5:131817300-131826465	21.8832	10.1904	1.102613038	5.00E-05	4.33E-03
IER5	NM_016545	chr1:181057637-181059979	24.8499	11.6178	1.096903147	5.00E-05	0.00433264
IRS1	NM_005544	chr2:227596032-227663506	3.97423	1.86159	1.094140002	5.00E-05	4.33E-03
C8orf4	NM_020130	chr8:40010986-40012827	79.8002	37.4443	1.091646243	5.00E-05	0.00433264
SLC16A12	NM_213606	chr10:91190050-91295313	38.3988	18.026	1.09098193	5.00E-05	0.00433264
TOB2	NM_016272	chr22:41829491-41843027	51.3603	24.1903	1.086224968	5.00E-05	0.00433264
AMOTL2	NM_016201	chr3:134074186-134094259	39.8798	18.8862	1.078325721	5.00E-05	0.00433264
RGS2	NM_002923	chr1:192778168-192781407	20.901	9.92095	1.075021789	2.50E-04	0.017201
PPP1R16B	NM_015568	chr20:37434347-37551667	28.2809	13.4484	1.072393492	5.00E-05	0.00433264
MESDC1	NM_022566	chr15:81293294-81296345	28.7209	13.6853	1.069473897	5.00E-05	0.00433264
EPHA2	NM_004431	chr1:16450831-16482582	13.3581	6.36869	1.068646265	5.00E-05	0.00433264

AGXT	NM_000030	chr2:241808161-241818536	24.6101	11.7356	1.068358916	3.50E-04	0.0227226
ZFP36L2	NM_006887	chr2:43449540-43453745	73.2467	34.9801	1.066229349	5.00E-05	0.00433264
CYP4A11	NM_000778	chr1:47394845-47407156	326.809	156.126	1.065736902	1.00E-04	0.00787453
PLIN2	NM_001122	chr9:19115758-19127604	130.436	62.3504	1.064871384	5.00E-05	0.00433264
MYCN	NM_005378	chr2:16076386-16087129	9.20815	4.40177	1.064827571	3.50E-04	0.0227226
GRAMD1B	NM_001286564	chr11:123396343-123498479	13.6515	6.57639	1.053691717	5.00E-05	0.00433264
PIM3	NM_001001852	chr22:50354142-50357720	114.346	55.219	1.050169232	5.00E-05	0.00433264
SPSB1	NM_025106	chr1:9352940-9429590	9.03553	4.38454	1.043183737	3.50E-04	0.0227226
PER2	NM_022817	chr2:239152678-239197207	3.52091	1.70869	1.043057671	2.50E-04	0.017201
EFNA1	NM_004428	chr1:155100348-155107386	69.9564	34.0428	1.039106346	5.00E-05	0.00433264
HES1	NM_005524	chr3:193853930-193856401	110.783	54.8973	1.012929412	5.00E-05	0.00433264
JMJD6	NM_015167	chr17:74708913-74722881	22.8972	11.3807	1.008581891	5.00E-04	0.0306361
BTNL9	NM_152547	chr5:180467224-180488523	12.2148	6.07672	1.007265519	5.00E-05	0.00433264
PLCH2	NM_014638	chr1:2407753-2436964	21.7662	10.8778	1.000702754	5.00E-05	0.00433264
GPR98	NR_003149	chr5:89854616-90460033	1.69738	0.869411	0.965199329	5.50E-04	0.0327313
SIM2	NM_005069	chr21:38071990-38122510	6.2941	3.24039	0.95783264	7.00E-04	0.0401579
SERTAD3	NM_203344	chr19:40946747-40950282	48.6484	25.151	0.951776594	1.00E-04	0.00787453
KBTBD11	NM_014867	chr8:1922043-1955109	16.5916	8.62267	0.944246446	5.00E-05	0.00433264
PLAU	NM_002658	chr10:75669726-75682535	38.79	20.21	0.940823216	1.00E-04	7.87E-03
MST1L	NM_001271733	chr1:17081128-17090975	21.7529	11.3353	0.940385174	5.00E-05	0.00433264
SLC20A1	NM_005415	chr2:113403433-113421400	19.9826	10.4512	0.935075708	1.00E-04	0.00787453
PPTC7	NM_139283	chr12:110972236-111021064	8.10309	4.23889	0.934785636	4.50E-04	0.0281556
ID4	NM_001546	chr6:19837600-19842431	23.3247	12.2087	0.933948937	5.00E-05	0.00433264
SUSD3	NM_145006	chr9:95820969-95847418	34.3588	18.1101	0.923885138	6.50E-04	0.0378132
MKNK2	NM_199054	chr19:2037469-2051243	66.1042	34.9264	0.920423992	5.00E-05	0.00433264

TNFRSF21	NM_014452	chr6:47199262-47277683	68.68	36.37	0.916870193	5.00E-05	4.33E-03
SNHG5	NR_003038	chr6:86386724-86388451	91.6959	48.6664	0.91393117	7.00E-04	0.0401579
G0S2	NM_015714	chr1:209848669-209849735	231.082	122.857	0.911424826	1.00E-04	0.00787453
KDM6B	NM_001080424	chr17:7743234-7758118	19.6511	10.4477	0.911424695	5.00E-05	0.00433264
FZD5	NM_003468	chr2:208627309-208634143	10.0295	5.33407	0.910941022	1.00E-04	7.87E-03
PTP4A1	NM_003463	chr6:64281916-64293493	23.0313	12.2762	0.907731791	5.00E-05	0.00433264
ETNK2	NM_018208	chr1:204100189-204121307	139.625	75.1306	0.894084752	1.00E-04	0.00787453
STK32B	NM_018401	chr4:5053526-5502725	11.3293	6.10208	0.892685725	3.50E-04	0.0227226
ID2	NM_002166	chr2:8822112-8824583	152.83	82.6228	0.88731591	1.50E-04	0.0111745
SOWAHC	NM_023016	chr2:110371910-110376564	9.90027	5.35336	0.887023197	3.00E-04	0.0198498
IFRD1	NM_001550	chr7:112063198-112117258	12.3207	6.66572	0.886251603	7.50E-04	0.0424385
BCL3	NM_005178	chr19:45251977-45263301	29.3818	15.9509	0.881284954	4.00E-04	0.025411
TP53INP2	NM_021202	chr20:33292147-33301237	41.2082	22.4181	0.878267437	1.00E-04	0.00787453
RASSF10	NM_001080521	chr11:13030969-13033653	11.4802	6.26248	0.87434178	5.50E-04	0.0327313
HRG	NM_000412	chr3:186383746-186396023	36.3002	19.8074	0.873937978	2.00E-04	0.0143819
IRF2BPL	NM_024496	chr14:77490885-77495042	48.2996	26.3989	0.871533425	2.00E-04	0.0143819
ARL4D	NM_001661	chr17:41476352-41478504	50.9311	27.8618	0.870258411	2.00E-04	0.0143819
CTGF	NM_001901	chr6:132269316-132272518	160.89	89.07	0.853009592	4.50E-04	2.82E-02
CLDN16	NM_006580	chr3:190105660-190129932	19.9491	11.141	0.840444928	2.50E-04	0.017201
ICAM1	NM_000201	chr19:10381516-10397291	23.6825	13.2305	0.8399538	5.50E-04	3.27E-02
PHLDA1	NM_007350	chr12:76419226-76425556	18.3245	10.3166	0.828806248	3.00E-04	0.0198498
CX3CL1	NM_002996	chr16:57406413-57418956	56.407	31.8731	0.823534866	2.50E-04	0.017201
IFIT1	NM_001548	chr10:91152302-91166244	15.4506	8.74992	0.820321132	5.50E-04	0.0327313
CNKSR3	NM_173515	chr6:154726432-154831753	18.9167	10.743	0.816263509	3.50E-04	0.0227226
LATS2	NM_014572	chr13:21547175-21635722	10.4978	5.99302	0.808731924	5.50E-04	0.0327313

SORD	NM_003104	chr15:45315301-45367287	101.393	57.9201	0.807822056	4.00E-04	0.025411
ABTB2	NM_145804	chr11:34172533-34379555	8.59975	4.92421	0.804402431	5.00E-04	0.0306361
CALML3	NM_005185	chr10:5566923-5568225	204.965	117.689	0.800398092	9.00E-04	0.0487932
ADAMTS1	NM_006988	chr21:28208605-28217728	23.0482	13.2347	0.800328592	5.50E-04	0.0327313
EMP1	NM_001423	chr12:13349601-13369708	22.8918	13.2697	0.786695153	8.50E-04	0.046325
SLC31A2	NM_001860	chr9:115913237-115926422	26.3922	15.4486	0.772635513	7.00E-04	0.0401579
PPARGC1A	NM_013261	chr4:23793643-23891700	18.3591	10.7976	0.765784659	7.50E-04	4.24E-02
PITPNM2	NM_020845	chr12:123468026-123594975	12.7427	7.60128	0.745356713	8.00E-04	0.0441813
KLF15	NM_014079	chr3:126061477-126076236	53.4183	31.89	0.744229966	5.50E-04	0.0327313
BTG1	NM_001731	chr12:92378751-92539673	33.0547	20.077	0.71931171	6.50E-04	0.0378132
ETS2	NM_005239	chr21:40177230-40196878	40.7526	24.8125	0.715825003	9.00E-04	0.0487932
RAMP3	NM_005856	chr7:45197366-45223850	53.0411	91.4573	-0.785987633	8.00E-04	0.0441813
TPM2	NM_213674	chr9:35681989-35690053	49.3846	87.3246	-0.822326905	8.00E-04	0.0441813
GSTT1	NM_000853	chr22:24376138-24384284	90.0251	160.236	-0.83179911	8.00E-04	0.0441813
COL6A2	NM_001849	chr21:47518032-47552763	18.8794	33.7886	-0.839723659	8.50E-04	0.046325
FKBP10	NM_021939	chr17:39968961-39979469	6.36256	11.4266	-0.84471693	6.50E-04	0.0378132
MFGE8	NM_005928	chr15:89441913-89456663	15.42	27.7383	-0.847076607	4.00E-04	0.025411
BST2	NM_004335	chr19:17513747-17516457	96.1866	173.765	-0.853229694	3.00E-04	0.0198498
APLNR	NM_005161	chr11:57001051-57004927	10.7525	19.4305	-0.853650895	2.00E-04	0.0143819
CSTB	NM_000100	chr21:45193545-45196256	54.5056	98.6804	-0.856359101	1.50E-04	0.0111745
ANXA2	NM_004039	chr15:60639349-60690185	58.2173	107.241	-0.88133674	7.00E-04	0.0401579
PSMB10	NM_002801	chr16:67968406-67970780	22.3215	41.3515	-0.889505684	3.00E-04	0.0198498
NPHS2	NM_014625	chr1:179334854-179545084	106.262	196.928	-0.890042482	5.00E-04	0.0306361
VIM	NM_003380	chr10:17256237-17279592	123.957	231.159	-0.899045789	3.00E-04	0.0198498
ARHGDIB	NM_001175	chr12:15094949-15114562	45.9355	86.0815	-0.906093685	5.00E-05	0.00433264

CHI3L1	NM_001276	chr1:203148058-203155922	22.092	42.0743	-0.929415237	1.50E-04	0.0111745
CFH	NM_000186	chr1:196621007-196716634	3.31	6.31	-0.930546756	4.50E-04	2.82E-02
KLK6	NM_001012964	chr19:51461886-51472929	16.4487	31.4611	-0.935595547	5.00E-05	0.00433264
RNASE1	NM_002933	chr14:21269514-21271036	174.076	337.546	-0.955366812	1.50E-04	0.0111745
MARCKS	NM_002356	chr6:114178526-114184652	7.52716	14.6076	-0.956541622	5.00E-05	0.00433264
S100A11	NM_005620	chr1:152004981-152009511	145.636	282.691	-0.956858931	1.00E-04	0.00787453
SERPING1	NM_001032295	chr11:57365026-57382326	57.7892	114.393	-0.985126969	2.50E-04	0.017201
RARRES3	NM_004585	chr11:63014620-63330855	58.6897	116.547	-0.98973263	5.50E-04	0.0327313
IFITM1	NM_003641	chr11:313990-315272	94.9927	192.531	-1.019202203	5.00E-05	0.00433264
PCOLCE	NM_002593	chr7:100183955-100205798	7.54891	15.3907	-1.027718598	5.00E-04	0.0306361
IFI27L2	NM_032036	chr14:94594117-94595957	39.6634	82.085	-1.049310259	1.50E-04	0.0111745
CDH6	NM_004932	chr5:31193761-31329253	7.16765	14.876	-1.053414556	5.00E-05	0.00433264
C2orf74	NM_001143960	chr2:61372242-61391964	17.584	36.7793	-1.064630731	5.00E-05	0.00433264
CAPN3	NM_173089	chr15:42651697-42704515	41.6599	87.3187	-1.067631275	1.00E-04	0.00787453
IFI6	NM_002038	chr1:27992571-27998724	43.6403	91.8617	-1.073802463	1.00E-04	0.00787453
HAVCR2	NM_032782	chr5:156512842-156536248	18.93	40.44	-1.095206532	5.00E-05	4.33E-03
RP9	NM_203288	chr7:33134409-33149002	9.89861	21.2655	-1.103216921	4.00E-04	0.025411
STRIP2	NM_020704	chr7:129074273-129128239	3.48821	7.50928	-1.10618769	5.00E-05	0.00433264
HCLS1	NM_005335	chr3:121350245-121379791	4.39477	9.46572	-1.106924583	6.50E-04	0.0378132
PSMB9	NM_002800	chr6_ssto_hap7:4252710-4258398	16.65	36.05	-1.114753137	2.50E-04	1.72E-02
PKI55	NR_037701	chr2:217081611-217084915	2.40196	5.23802	-1.124809442	3.00E-04	0.0198498
VWF	NM_000552	chr12:6058039-6233836	1.19646	2.65236	-1.148504438	1.50E-04	1.12E-02
TNC	NM_002160	chr9:117781853-117880536	2.92431	6.5047	-1.153386264	5.00E-05	0.00433264
CLU	NM_001831	chr8:27454433-27472328	112.371	250.905	-1.158871459	1.50E-04	1.12E-02
APOL1	NM_003661	chr22:36649116-36663577	16.2135	36.2324	-1.160084813	5.00E-05	0.00433264

HOXC8	NM_022658	chr12:54402889-54406545	3.38577	7.69048	-1.183589674	3.00E-04	0.0198498
LTC4S	NM_145867	chr5:179220985-179223513	10.1991	23.3321	-1.193874313	7.50E-04	4.24E-02
MAP1B	NM_005909	chr5:71403117-71505397	2.79901	6.49462	-1.214328475	5.00E-05	0.00433264
PROM1	NM_001145848	chr4:15969848-16085623	2.94867	6.8682	-1.219867678	5.00E-05	0.00433264
S100A4	NM_002961	chr1:153516094-153518282	35.3856	83.8205	-1.244140745	1.00E-04	0.00787453
ECM1	NM_004425	chr1:150480486-150486265	5.12396	12.1643	-1.247322184	5.00E-05	0.00433264
ADH1B	NM_000668	chr4:100227543-100242599	2.29235	5.50426	-1.263721285	2.00E-04	0.0143819
IGFBP6	NM_002178	chr12:53491435-53496128	9.94096	24.2694	-1.287681357	5.00E-05	0.00433264
LOC100216546	NR_039981	chr7:104622193-104631612	1.40663	3.43879	-1.289658124	5.00E-05	0.00433264
MDK	NM_002391	chr11:46402333-46405387	10.2069	25.2125	-1.304594416	2.50E-04	1.72E-02
MYO1F	NM_012335	chr19:8585673-8642331	0.986361	2.49806	-1.340620466	6.00E-04	0.0356046
MMP2	NM_004530	chr16:55513080-55540586	3.39468	8.6244	-1.345148507	5.00E-05	4.33E-03
AEBP1	NM_001129	chr7:44143959-44154164	13.8324	35.1496	-1.345456778	5.00E-05	0.00433264
PTN	NM_002825	chr7:136912091-137028546	4.35039	11.0907	-1.35013378	1.00E-04	0.00787453
ISG15	NM_005101	chr1:948846-949919	18.9095	48.3431	-1.354198772	5.00E-05	0.00433264
HSPB6	NM_144617	chr19:36245466-36247930	5.52193	14.2387	-1.366572929	5.00E-05	0.00433264
LGALS1	NM_002305	chr22:38071612-38075809	129.247	335.16	-1.374719185	5.00E-05	0.00433264
RAMP1	NM_005855	chr2:238768186-238820755	4.10642	10.6606	-1.376335541	8.00E-04	0.0441813
HNF1A-AS1	NR_024345	chr12:121407640-121410095	8.28221	21.5033	-1.376470391	5.00E-05	0.00433264
WFDC2	NM_006103	chr20:44098393-44110172	143.676	374.156	-1.38082082	5.00E-05	0.00433264
ENO2	NM_001975	chr12:7023613-7032859	3.25169	8.55904	-1.396259265	5.00E-05	0.00433264
HBA2	NM_000517	chr16:222845-223709	14.9284	39.4605	-1.402349688	4.00E-04	0.025411
ANKRD36B	NM_025190	chr2:98121260-98206428	0.558945	1.49785	-1.42211492	7.50E-04	0.0424385
MGP	NM_000900	chr12:15034114-15038853	74.5418	201.432	-1.43417133	5.00E-05	0.00433264
C2orf40	NM_032411	chr2:106682112-106694609	12.8145	35.057	-1.451925355	1.00E-04	0.00787453

PLAC9	NM_001012973	chr10:81892257-81904784	5.07753	14.0488	-1.468248142	8.50E-04	0.046325
CRIP1	NM_001311	chr14:105953256-105955124	28.3434	79.8605	-1.494469274	5.00E-05	0.00433264
CFB	NM_001710	chr6_ssto_hap7:3246430-3252571	13.6431	38.5522	-1.498641698	5.00E-05	0.00433264
LOXL4	NM_032211	chr10:100007442-100028007	0.869817	2.46628	-1.503552789	3.50E-04	0.0227226
LAPTM5	NM_006762	chr1:31205314-31230683	6.58805	18.7091	-1.50581675	5.00E-05	0.00433264
EMILIN1	NM_007046	chr2:27301434-27309265	5.3488	15.457	-1.530973173	5.00E-05	0.00433264
CYGB	NM_134268	chr17:74523429-74541458	1.70789	5.03529	-1.559859812	2.50E-04	0.017201
SPON2	NM_012445	chr4:1160720-1202750	12.1955	35.9744	-1.560621717	5.00E-05	0.00433264
CD44	NM_001001391	chr11:35160416-35253949	1.72	5.09	-1.562752414	4.00E-04	2.54E-02
LOC338799	NR_002809	chr12:122233172-122241390	5.36658	15.8682	-1.564063597	5.00E-05	0.00433264
NEGR1	NM_173808	chr1:71868624-72748277	0.869881	2.58537	-1.571480805	5.00E-05	0.00433264
IFI44L	NM_006820	chr1:79086087-79111830	1.76216	5.25578	-1.576559964	5.00E-05	0.00433264
NFAM1	NM_145912	chr22:42776413-42828401	0.414696	1.24371	-1.58452409	6.50E-04	0.0378132
CFD	NM_001928	chr19:859664-863610	1.71811	5.16717	-1.588551943	8.00E-04	0.0441813
OLFML3	NM_020190	chr1:114522012-114524876	2.67622	8.08953	-1.595859166	5.00E-05	0.00433264
COL14A1	NM_021110	chr8:121137346-121384273	0.395479	1.21967	-1.624817863	2.00E-04	0.0143819
ITGAX	NM_000887	chr16:31366454-31394318	0.64	1.97	-1.625567777	2.00E-04	1.44E-02
CTSS	NM_004079	chr1:150702671-150738433	1.90045	5.8656	-1.625937622	5.00E-05	0.00433264
ANKRD36	NM_001164315	chr2:97779232-97930257	0.559081	1.72885	-1.62868348	1.50E-04	0.0111745
TGFBI	NM_000358	chr5:135364583-135399507	4.3524	13.4904	-1.63205007	5.00E-05	0.00433264
LYZ	NM_000239	chr12:69742133-69748013	8.26676	25.6583	-1.634031679	5.00E-05	0.00433264
COL1A2	NM_000089	chr7:94023872-94060544	4.46972	14.166	-1.664176084	5.00E-05	4.33E-03
TSHZ2	NM_173485	chr20:51588945-52111869	0.367369	1.16528	-1.665374859	5.00E-05	0.00433264
RAC2	NM_002872	chr22:37621300-37640339	2.1904	7.10556	-1.697754003	1.00E-04	0.00787453
CD53	NM_000560	chr1:111413820-111442558	2.12248	6.93133	-1.707381247	5.00E-05	0.00433264

SPINK1	NM_003122	chr5:147204142-147211260	21.578	73.2411	-1.763092307	5.00E-05	0.00433264
APOBR	NM_018690	chr16:28505969-28510291	0.471979	1.66881	-1.822025132	2.50E-04	0.017201
SERPINF1	NM_002615	chr17:1665258-1680859	6.87096	24.5726	-1.838466926	5.00E-05	0.00433264
CX3CR1	NM_001337	chr3:39304984-39323226	0.592977	2.13185	-1.846057879	3.00E-04	0.0198498
CCL5	NM_002985	chr17:34198495-34207377	2.34019	8.47198	-1.856073517	1.00E-04	0.00787453
C1R	NM_001733	chr12:7187514-7245043	10.2625	37.6556	-1.875482211	5.00E-05	0.00433264
APOC3	NM_000040	chr11:116700623-116703787	21.0174	77.428	-1.881271168	5.00E-05	4.33E-03
PTPRC	NM_080921	chr1:198608097-198726605	0.565205	2.08418	-1.882633748	1.50E-04	0.0111745
CORO1A	NM_007074	chr16:30194730-30200575	2.85401	10.7192	-1.909134943	5.00E-05	0.00433264
IL7R	NM_002185	chr5:35856976-35879705	0.399079	1.50509	-1.915103489	1.50E-04	1.12E-02
CCDC88B	NM_032251	chr11:64107689-64125006	0.41498	1.59677	-1.944042808	5.00E-05	0.00433264
SLC37A2	NM_001145290	chr11:124933012-124960412	0.299997	1.16301	-1.954843523	5.00E-04	0.0306361
C7	NM_000587	chr5:40909598-40983042	14.9406	58.1232	-1.959876047	5.00E-05	0.00433264
PLD4	NM_138790	chr14:105391186-105399573	0.581727	2.27209	-1.965605811	8.50E-04	0.046325
TUBB3	NM_006086	chr16:89988416-90002505	1.16378	4.61188	-1.986536617	2.00E-04	0.0143819
RMRP	NR_003051	chr9:35657747-35658015	53.5594	215.692	-2.009760964	5.00E-05	0.00433264
TFPI	NM_006287	chr2:188328957-188419219	11.0155	44.9857	-2.029931491	5.00E-05	0.00433264
TYROBP	NM_003332	chr19:36395302-36399211	6.80985	28.1838	-2.049171217	3.00E-04	0.0198498
COL3A1	NM_000090	chr2:189839098-189877472	4.89921	20.2881	-2.050012724	5.00E-05	0.00433264
LAMC3	NM_006059	chr9:133884503-133968446	0.591846	2.45982	-2.055259012	5.00E-05	0.00433264
APOA1	NM_000039	chr11:116706468-116708338	2.94095	12.4123	-2.07741631	5.00E-05	4.33E-03
LCP1	NM_002298	chr13:46700057-46756459	1.8945	8.04341	-2.085990121	5.00E-05	0.00433264
FBLN1	NM_001996	chr22:45898718-45997014	0.905935	3.8509	-2.087716213	6.50E-04	0.0378132
LAMC2	NM_005562	chr1:183155173-183214262	0.772712	3.38899	-2.132852672	5.00E-05	0.00433264
CYTH4	NM_013385	chr22:37678494-37711389	0.73463	3.23867	-2.140311757	5.00E-05	0.00433264

C1QC	NM_172369	chr1:22970117-22974603	8.25925	36.5244	-2.144777888	5.00E-05	0.00433264
C1QA	NM_015991	chr1:22963117-22966175	8.57	38.14	-2.154125652	5.00E-05	4.33E-03
NNMT	NM_006169	chr11:114166534-114183238	1.91018	8.52962	-2.158772877	5.00E-05	0.00433264
LSP1	NM_002339	chr11:1874199-1913493	2.23407	9.98945	-2.160730858	5.00E-05	0.00433264
CYBB	NM_000397	chrX:37639269-37672714	1.11949	5.05848	-2.1758623	5.00E-05	0.00433264
CSF1R	NM_001288705	chr5:149432853-149492935	1.44982	6.56714	-2.179391415	3.00E-04	0.0198498
MOXD1	NM_015529	chr6:132617193-132722664	0.690171	3.14798	-2.189400615	5.00E-05	0.00433264
COL16A1	NM_001856	chr1:32117847-32169768	0.431505	2.0151	-2.223402253	5.00E-05	0.00433264
S100A1	NM_006271	chr1:153591275-153618782	52.2033	250.257	-2.26119751	1.00E-04	0.00787453
TIMP1	NM_003254	chrX:47420498-47479256	28.7327	140.69	-2.291754314	7.50E-04	0.0424385
PTGIR	NM_000960	chr19:47123724-47128354	0.640264	3.1682	-2.306924613	4.50E-04	2.82E-02
APOC1	NM_001645	chr19:45417920-45422606	6.16172	30.7144	-2.317510171	5.00E-05	0.00433264
CD3E	NM_000733	chr11:118175294-118186890	0.575887	2.88296	-2.32369316	8.50E-04	0.046325
MMP7	NM_002423	chr11:102391238-102401478	27.1193	145.989	-2.428467823	5.00E-05	4.33E-03
PLEK	NM_002664	chr2:68592321-68624585	0.527834	2.86584	-2.440801877	5.00E-05	0.00433264
OLFM4	NM_006418	chr13:53602875-53626196	1.45473	7.94003	-2.448393047	5.00E-05	0.00433264
TPSAB1	NM_003294	chr16:1290677-1292555	0.695247	3.84262	-2.466492795	7.00E-04	0.0401579
RPPH1	NR_002312	chr14:20811229-20811570	27.0056	151.029	-2.48349509	5.00E-05	0.00433264
SASH3	NM_018990	chrX:128913891-128929176	0.497597	2.82543	-2.505420754	1.50E-04	0.0111745
FCER1G	NM_004106	chr1:161185086-161189038	3.98363	23.3558	-2.551625301	5.00E-05	0.00433264
C1QB	NM_000491	chr1:22979681-22988029	4.99	29.42	-2.558667284	5.00E-05	4.33E-03
FBLN1	NM_006486	chr22:45898718-45997014	1.34777	8.01816	-2.572696888	5.00E-05	0.00433264
IL2RG	NM_000206	chrX:70327253-70331481	0.717954	4.45805	-2.63444948	5.00E-05	4.33E-03
CCL21	NM_002989	chr9:34709001-34710164	3.37686	21.1192	-2.644800913	5.00E-05	0.00433264
CD48	NM_001778	chr1:160648535-160681641	0.880747	5.68054	-2.68922852	3.00E-04	0.0198498

TMEM119	NM_181724	chr12:108983621-108991894	0.347514	2.29486	-2.723263139	1.50E-04	0.0111745
SLPI	NM_003064	chr20:43880879-43883205	4.6341	35.5033	-2.937592046	5.00E-05	0.00433264
FPR3	NM_002030	chr19:52298410-52329334	0.229123	1.83803	-3.003966121	8.00E-04	0.0441813
MMP9	NM_004994	chr20:44637546-44645200	0.33	2.68	-3.031835525	1.50E-04	1.12E-02
LUM	NM_002345	chr12:91497231-91505542	2.97781	25.5171	-3.099140767	5.00E-05	0.00433264
MIR3648	NR_037421	chr21:9825831-9826011	203.431	1850.82	-3.185553147	5.00E-05	0.00433264
C1S	NM_001734	chr12:7167979-7178335	0.985587	9.28092	-3.235212692	2.00E-04	0.0143819
COL1A1	NM_000088	chr17:48261456-48279000	1.71498	16.2487	-3.244060641	5.00E-05	4.33E-03
COL6A3	NM_057166	chr2:238232654-238322850	0.319486	3.60237	-3.49512175	5.00E-05	0.00433264
IGJ	NM_144646	chr4:71521257-71532348	1.18791	13.9709	-3.55592752	5.00E-05	0.00433264
C3	NM_000064	chr19:6677845-6720662	2.09068	26.5683	-3.667661754	5.00E-05	0.00433264
SERPINA3	NM_001085	chr14:95078713-95090390	1.33363	21.6808	-4.022987623	5.00E-05	0.00433264
REN	NM_000537	chr1:204123943-204135465	10.2196	299.817	-4.874671553	5.00E-05	0.00433264

Table S2. Genes and proteins related with IgA nephropathy.

Genes and proteins listed in the table are searched through the Pubmed database and were previously described to be related with IgAN. Gene name and protein name are given, reference papers are also listed.

Number	Title	Genes	Proteins
1	Variants in Complement Factor H and Complement Factor H-Related Protein Genes, CFHR3 and CFHR1, Affect Complement Activation in IgA Nephropathy	CFH, CFHR3, and CFHR1	
2	Association of Megsin gene polymorphism with IgA nephropathy risk	Megsin	
3	Association of angiotensin II type-1 receptor A1166C gene polymorphism with the susceptibility of immunoglobulin A nephropathy	angiotensin II type-1 receptor (AT1R) A1166C	
4	Association between plasminogen activator inhibitor-1 4G/5G gene polymorphism and immunoglobulin A nephropathy susceptibility	plasminogen activator inhibitor-1 (PAI-1) 4G/5G	
5	Nephrokeli, a Chinese herbal formula, may improve IgA nephropathy through regulation of the sphingosine-1-phosphate pathway		CTGF,S1PR2 and S1PR3
6	BAFF promotes proliferation of human mesangial cells through interaction with BAFF-R	BIM	BAFF,NF-kappaBp65, Akt and MAPK p38 kinase
7	Urinary CXCL1: a novel predictor of IgA nephropathy progression		CXCL1
8	NFkappaB upregulates ubiquitin Cterminal hydrolase 1 in diseased podocytes in glomerulonephritis		NFkappaB,UCHL1
9	IL-17A as a potential biomarker of IgA nephropathy		IL-17a
10	Intermedin ameliorates IgA nephropathy by inhibition of oxidative stress and inflammation		Intermedin,NF-kappa B p65p65,TGFbeta-1,collage n IV,inflammatory factors (TNF-alpha, MCP-1 and MMP-9)
11	Changes in urinary angiotensinogen posttreatment in pediatric IgA nephropathy		urinary AGT

	patients		
12	Role of tubulointerstitial plasmin in the progression of IgA nephropathy		plasmin
13	CD147 renal expression as a biomarker for progressive IgAN		CD147
14	DNA methylation in Cosmc promoter region and aberrantly glycosylated IgA1 associated with pediatric IgA nephropathy		hypermethylation of Cosmc promoter region
15	N-acetylgalactosaminide alpha2,6-sialyltransferase II is a candidate enzyme for sialylation of galactose-deficient IgA1, the key autoantigen in IgA nephropathy		ST6GalNAc-II
16	Circulating TNF receptors 1 and 2 are associated with the severity of renal interstitial fibrosis in IgA nephropathy		TNFR
17	Imbalance of interleukin-18 and interleukin-18 binding protein in patients with IgA nephropathy implicating renal vasculopathy		IL-18/IL-18BP
18	Identification of mannose-binding lectin as a mechanism in progressive immunoglobulin A nephropathy		
19	Role of let-7b in the regulation of N-acetylgalactosaminyltransferase 2 in IgA nephropathy		GALNT2
20	TWEAK/Fn14 system and crescent formation in IgA nephropathy		
21	Inhibition of SET Domain-Containing Lysine Methyltransferase 7/9 Ameliorates Renal Fibrosis		SET7/9
22	Diagnostic urinary proteome profile for immunoglobulin a nephropathy		CD44, glycoprotein 2, vasorin, epidermal growth factor, CLM9, protocadherin, utreoglobin, dipeptidyl peptidase IV, NHL repeat-containing protein 3, and SLAM family

			member 5
23	High serum and urine neutrophil gelatinaseassociated lipocalin levels are independent predictors of renal progression in patients with immunoglobulin A nephropathy		neutrophil gelatinase-associated lipocalin (NGAL)
24	DEFA gene variants associated with IgA nephropathy in a Chinese population	DEFA	
25	Plasma neutrophil gelatinase-associated lipocalin as a potential predictor of adverse renal outcomes in immunoglobulin A nephropathy		NGAL
26	Gluten exacerbates IgA nephropathy in humanized mice through gliadin-CD89 interaction		gluten,gliadin-CD89
27	Absence of mesangial c1q deposition is associated with resolution of proteinuria and hematuria after tonsillectomy plus steroid pulse therapy for immunoglobulin a nephropathy		c1q
28	Association of glomerular C4d deposition with various demographic data in IgA nephropathy patients; a preliminary study		c4d
29	A SPRY2 mutation leading to MAPK/ERK pathway inhibition is associated with an autosomal dominant form of IgA nephropathy	SPRY2	
30	Decreased expression of follicular dendritic cell-secreted protein correlates with increased immunoglobulin A production in the tonsils of individuals with immunoglobulin A nephropathy	FDC-SP	
31	Correlation of disease activity in proliferative glomerulonephritis with glomerular spleen tyrosine kinase expression	SYK	
32	Neutrophil gelatinase-associated lipocalin as a predictor of adverse renal outcomes in immunoglobulin A nephropathy		NGAL
33	Association of uteroglobin G38A gene polymorphism with IgA nephropathy risk: an updated meta-analysis	G38A	uteroglobin G38A
34	Implication of urinary complement factor h in the progression of immunoglobulin a	Urinary CFH	

	nephropathy		
35	Identification of new susceptibility loci for IgA nephropathy in Han Chinese	ST6GAL1,ACCS ,ODF1-KLF10, ITGAX-ITGAM,DEFA,	
36	Prostacyclin synthase: upregulation during renal development and in glomerular disease as well as its constitutive expression in cultured human mesangial cells		PGI2
37	Toll-like receptor 4 is involved in a protective effect of rhein on immunoglobulin A nephropathy		TLR4, TGF-beta1,
38	Brief Report: identification of MTMR3 as a novel susceptibility gene for lupus nephritis in northern Han Chinese by shared-gene analysis with IgA nephropathy	MTMR3	
39	Association of systemic lupus erythematosus susceptibility genes with IgA nephropathy in a Chinese cohort	CFH,HLA-DRA,HLA-DRB1,PXK,BLK,UBE2L3	
40	Association of T869, C509T, G915C gene polymorphism of transforming growth factor-beta1 with IgA nephropathy risk	T869, C509T, G915C in TGFbeta1	
41	Association of aldosterone synthase (CYP11B2) gene polymorphism with IgA nephropathy risk and progression of IgA nephropathy	aldosterone synthase (CYP11B2)	
42	Elevated soluble VEGF receptor sFlt-1 correlates with endothelial injury in IgA nephropathy	sFlt-1	
43	Expression of human T cell immunoglobulin domain and mucin-3 on kidney tissue from immunoglobulin A nephropathy patients		Tim-3
44	CagA, a major virulence factor of Helicobacter pylori, promotes the production and underglycosylation of IgA1 in DAKIKI cells		CagA,C1GALT1
45	Expression of age-related factors during the development of renal damage in patients with IgA nephropathy		8-OHdG,p16,Klotho,
46	Polymorphism of DEFA in Chinese Han population with IgA nephropathy	DEFA	
47	Urinary kidney injury molecule-1 is related to pathologic involvement in IgA		kidney injury molecule-1

	nephropathy with normotension, normal renal function and mild proteinuria		(KIM-1)
48	N-acetylgalactosamine exposure of serum IgA1 was associated with glomerulosclerosis and tubular atrophy/interstitial fibrosis of IgA nephropathy patients		GalNAc
49	Effect of Eclipta prostrata on 11Beta-Hydroxysteroid Dehydrogenase in Rat Liver and Kidney		11 beta -HSD
50	Reduced mir-29b-3p expression up-regulate CDK6 and contributes to IgA nephropathy		CDK6
51	Renal expression of advanced oxidative protein products predicts progression of renal fibrosis in patients with IgA nephropathy		AOPPs
52	Univariate and multiple linear regression analyses for 23 single nucleotide polymorphisms in 14 genes predisposing to chronic glomerular diseases and IgA nephropathy in Han Chinese	CTLA4,CR2	
53	Epithelial protein lost in neoplasm modulates platelet-derived growth factor-mediated adhesion and motility of mesangial cells		EPLIN
54	ACE insertion/deletion polymorphism (rs1799752) modifies the renoprotective effect of renin-angiotensin system blockade in patients with IgA nephropathy	ACE I/D	
55	Ectopic expression of fatty acid-binding protein 4 in the glomerulus is associated with proteinuria and renal dysfunction		FABPs
56	Cytokines alter IgA1 O-glycosylation by dysregulating C1GalT1 and ST6GalNAc-II enzymes		IL-6,IL-4
57	Capsaicin induces high expression of BAFF and aberrantly glycosylated IgA1 of tonsillar mononuclear cells in IgA nephropathy patients		BAFF,capsaicin,cosmc,galnac2,c1galt1
58	Significance of urinary full-length megalin in patients with IgA nephropathy		urinary C-megalin
59	The combined role of galactose-deficient IgA1 and streptococcal IgA-binding M Protein in inducing IL-6 and C3 secretion from human mesangial cells: implications for IgA nephropathy		streptococcal IgA-binding M protein
60	Association of aldosterone synthase (CYP11B2) gene -344T/C polymorphism with the	CYP11B2	

	risk of primary chronic glomerulonephritis in the Polish population		
61	Association of BH3 interacting domain death agonist (BID) gene polymorphisms with proteinuria of immunoglobulin A nephropathy	BID	
62	Association of FOS-like antigen 1 promoter polymorphism with podocyte foot process effacement in immunoglobulin A nephropathy patients	FOSL1	
63	liu 2014 nature	1q32 (CFHR3-CFHR1 genes), 6p21 (MHC),8p23 (DEFA gene cluster), 17p13.1 (TNFSF13) and 22q12(HORMAD2),	
64	Mapping Novel Immunogenic Epitopes in IgA nephropathy		ZADH2,GRINL1A,DDX4
65	Geographic differences in genetic susceptibility to IgA nephropathy: GWAS replication study and geospatial risk analysis	Chr.6p21 (HLA-DQB1/DRB1, PSMB9/TAP1, and DPA1/DPB2 loci),Chr.1q32 (CFHR3/R1 locus), and Chr.22q12 (HORMAD2 locus).	
66	Cellular Signaling and Production of Galactose-Deficient IgA1 in IgA Nephropathy, an Autoimmune Disease	HLA-DRB1, HLA-DQA1, HLA-DQB1	
		IL-6 related cytokines--LIF & OSM	
		PSMB8, PSMB9, TAP1, TAP2	
		HLA-DPB1, HLA-DPB2, HLA-DPA1, COL11A2	
		CFH, CFHR HORMAD2 locus	
		HORMAD2, MTMR3, LIF,	

		OSM	
		DEFA	
		TNFSF13 (APRIL)	
67	Advanced Oxidation Protein Products Activate Intrarenal Renin–Angiotensin System via a CD36-Mediated, Redox-Dependent Pathway		AOPPS
68	Urokinase, urokinase receptor, and plasminogen activator inhibitor-1 expression on podocytes in immunoglobulin A glomerulonephritis		PLAU
69	Tubular NF-kappaB and AP-1 activation in human proteinuric renal disease		NFkappaB,AP-1
70	Transforming growth factor-beta, endothelin-1, and c-fos expression in necrotizing/crescentic IgA glomerulonephritis		c-FOS
71	Cytokine mRNA expression by cultured rat mesangial cells after contact with environmental lectins	c-FOS	
72	Advanced oxidation protein products activate intrarenal renin-angiotensin system via a CD36-mediated, redox-dependent pathway		JUN
73	IgA induced activation of human mesangial cells: independent of FcalphaR1 (CD 89)		c-JUN
